# Whole-Genome, Recombinant, and Phylogenetic Analysis of Porcine Epidemic Diarrhea Virus Strain CH/JSXZ/2015

**DOI:** 10.1155/2023/2991270

**Published:** 2023-11-08

**Authors:** Changchao Huan, Xinyue Guo, Jinlong Cheng, Pengxiang Chen, Bo Ni, Wei Zhang, Jingting Yao, Luyao Jiang, Song Gao

**Affiliations:** ^1^Institutes of Agricultural Science and Technology Development, College of Veterinary Medicine, Yangzhou University, Yangzhou 225009, Jiangsu, China; ^2^Yangzhou University and Jiangsu Co-Innovation Center for Prevention and Control of Important Animal Infectious Diseases and Zoonoses, Yangzhou 225009, Jiangsu, China; ^3^Key Laboratory of Avian Bioproduct Development, Ministry of Agriculture and Rural Affairs, Yangzhou 225009, Jiangsu, China; ^4^China Animal Health and Epidemiology Center, Qingdao, China; ^5^Institutes of Agricultural Science and Technology Development, Yangzhou University, Yangzhou 225009, Jiangsu, China

## Abstract

**Background:**

Porcine epidemic diarrhea virus (PEDV) is an important pathogen causing highly contact infectious intestinal infections in pigs belonging to the family *Coronaviridae*, genus *Coronavirus*, which can cause porcine epidemic diarrhea (PED). Since 2010, outbreaks of PEDV variants have caused great economic losses to the swine industry worldwide. Our study will provide the basis for discovering the key points of PEDV variation and help in understanding the trend of popularity and evolution of PEDV in China.

**Methods:**

We amplify the complete PEDV CH/JSXZ/2015 genome sequence from naturally infected piglets in Xuzhou, Jiangsu Province, China, by RT-PCR. The comparative genome circle graph, characterization and phylogenetic analysis, and recombination in the PEDV CH/JSXZ/2015 and other PEDVs are analyzed by bioinformatics analysis software.

**Results:**

At the whole-genome level, CH/JSXZ/2015 showed the highest nucleotide identity (99.5%) with CH/GDZHDM/1401 strain (KR153326.1 and KX016034.1) and the lowest similarity (93.5%) to 85-7-mutant1 strain. CH/JSXZ/2015 S amino acid identity was 90.8%–99.8% compared with other strains. CH/JSXZ/2015 ORF3 amino acid identity was 91.2%–100.0% compared with other PEDV strains. Phylogenetic analysis based on the whole genome, S, and ORF3 revealed that CH/JSXZ /2015 is most genetically close to the CH/GDZHDM/1401 strain (KR153326.1 and KX016034.1) isolated in China in 2014. In addition, we revealed that the CH/GDZHDM/1401 (KX016034.1) strain, PEDV 1842/2016 ITA, and CH/JSXZ /2015 had recombination, and the position of recombination was 1–5530 and 28185–28544 bp. 5531–28184 bp of JSXZ are highly homologous to CH/GDZHDM/1401 (KX016034.1), while 1–5530 and 28185–28544 bp of JSXZ are highly homologous to 1842/2016 ITA. These results helped discover PEDV variation rules and provided a basis for preventing and controlling PEDV.

**Conclusions:**

Our study revealed that PEDV CH/JSXZ/2015 was a variant strain, the current epidemic strain in China. The CH/GDZHDM/1401 (KX016034.1) strain, PEDV 1842/2016 ITA (KY111278) and CH/JSXZ /2015 had recombination.

## 1. Introduction

Porcine epidemic diarrhea virus (PEDV) is an important pathogen causing highly contact infectious intestinal infections in pigs belonging to the family *Coronaviridae*, genus *Coronavirus*, which can cause porcine epidemic diarrhea (PED) [1]. PEDV mainly causes diarrhea in pigs and occasional vomiting after eating or milking [1]. The younger the age, the more severe the symptoms. Pigs born within a week often show severe dehydration symptoms and die 3–4 days after the onset of diarrhea, with a mortality rate of up to 50% and a maximum mortality rate of 100% [2]. The sick pigs are depressed, with reduced or no appetite. Weaned pigs and sows with mental atrophy and persistent diarrhea gradually recovered to normal after about 1 week [2]. PEDV infects a protease-rich environment, the epithelium of the small intestine, causing dehydration [3]. As a result, PEDV often causes diarrhea and systemic symptoms such as vomiting, fever, anorexia, and lethargy [4]. The disease is more severe in lactating piglets because of their increased susceptibility to dehydration, but outbreaks have also occurred in growing pigs and occasionally in adult pigs [5].

PEDV genome is a single-stranded positive-strand infectious RNA [5]. The pathogen was first identified as *Coronavirus* in Belgium in 1978, and the virus was also named PEDV (PEDV strain CV777) [6]. The complete genomic sequence of PEDV has 28,038 nucleotides (nt), a 5′ cap and 3′ polyadenylate tail, and at least seven open reading frames (ORFs). PEDV genes are Pol gene, S gene, ORF3 gene, E gene, M gene, and N gene in order from 5′end to 3′end [7]. Pol gene is mainly used to encode the RNA polymerase of replicase polyprotein lab, which is important in the early stage of virus infection [8]. The S protein mainly affects the immune response [8]. After the virus infects the host, it can recognize the target cells and promote the cell membrane fusion with the virus [9]. Therefore, the S protein is the main target that can effectively resist the *Coronavirus* [10]. If the S of the virus mutates, it is very likely to cause changes in the range of the host and the culture and virulence of tissue cells [11]. The ORF3 gene is used to encode the ORF3 protein, a nonstructural protein that directly determines the pathogenicity of the virus [12]. The E gene is used to encode the E protein, which is the smallest structural protein of PEDV. This protein is scattered on the envelope of PEDV, which can promote self-assembly and budding of the virus. M protein is a penetrating protein that plays an important role in virus assembly and budding [13]. At the same time, M protein is an important structural protein used to stimulate the body to produce immune protection [14]. As a major structural protein of PEDV, the main function of N protein is to form nucleocapsid. At the same time, the N protein is also involved in the replication and transcription of the virus [15].

In this study, we determined the complete genome sequences of viruses from tissues of diseased pigs in Xuzhou, Jiangsu, China, to study the diversity of PEDV. We compared these sequences with existing sequences while we analyzed PEDV CH/JSXZ/2015 for genetic variation with the full sequences of existing sequences, S and ORF3. In addition, we explored whether PEDV CH/JSXZ/2015 had the recombination. This experiment helps grasp the prevalence of PEDV and the genetic variation characteristics of PEDV and provides a theoretical basis for scientific prevention and control of PED.

## 2. Materials and Methods

### 2.1. Source of Material

One swine farm from Xuzhou, Jiangsu Province in China, had PED outbreaks, and we got clinical samples, such as fecal swabs, fecal samples, and intestines, to amplify the whole genome of PEDV. The complete genome sequence of the PEDV strain was CH/JSXZ/2015. GenBank number was MT625963. All the reagents are listed in Table 1.

### 2.2. PEDV Primer Design and Synthesis

To amplify the complete PEDV genome sequence, we designed primers targeting different genes based on the genome of strain CV777 (AF353511.1). These primers are shown in Table 2.

### 2.3. RNA Extraction and RT-PCR

The tissue grinding fluid was frozen and thawed three times and pelleted by centrifugation for 15 min at 12,000 rpm. The supernatant was collected and used to extract viral RNA. Following the instructions, we used Trizol reagent (Vazyme Biotech) to extract virus RNA. 1 *μ*g of total RNA to synthesize cDNA according to the instructions of the reverse transcription kit (TransScript First-Strand cDNA Synthesis SuperMix). Using cDNA as a template, PCR amplification using Taq enzyme. Add the following reagents in sequence to the PCR tube to establish a 25 *μ*l amplification reaction system: upstream primer (10 *μ*M) 1 *μ*l; downstream primer (10 *μ*M) 1 *μ*l; 2 × Taq Master Mix 12.5 *μ*l; DNA template 1 *μ*l; and sterilized ultrapure water 9.5 *μ*l.

Reaction parameters for PEDV whole genome amplification: 95°C predenaturation for 3 min; denaturation at 95°C for 15 s, annealing at 50°C for 15 s, extension at 72°C for 1 min, 30 cycles; at last, extension at 72°C for 5 min and store at 12°C. The PCR amplification products were identified by agarose gel electrophoresis.

### 2.4. Viral Genome Cloning and Sequencing

After recovering the target gene by gel, the following reaction solution was prepared in a microcentrifuge tube (total volume of 5 *μ*l): T-Vector pMD19 (Simple) 1 *μ*l; gum recycling products 1 *μ*l (0.1–0.3 pM); and sterilized ultrapure water 3 *μ*l.

Add an equal volume (5 *μ*l) of DNA Ligation Kit (code no. 6023) to the above DNA solution and mix thoroughly. 16°C reaction for 24 hr. Add the full amount (10 *μ*l) to 100 *μ*l of *Escherichia coli* receptor cells, mix gently, and place on ice for 30 min. After a water bath at 42°C for 90 s, quickly transfer to ice for 5 min. Add 890 *μ*l of LB medium and incubate at 37°C for 60 min. Apply 100 *μ*l of the transformation solution on an agar plate medium and incubate overnight at 37°C. Select white colonies for PCR validation.

Then, positive clones were sent to Nanjing Kinco Biotechnology Co., Ltd. for sequencing.

### 2.5. Comparative Genome Analyzed by BRIG

The comparative genome circle graph is drawn by the software BRIG (Blasting Image Generator) [16].

### 2.6. Sequence Analysis

Sequence data were assembled and analyzed using the DNAStar package (DNAStar Inc.). The Clustal W method was used to analyze multiple sequence comparisons (the MegAlign program). The neighbor-joining method was used to construct the phylogenetic tree. The nucleotide and amino acid sequences of PEDV were compared with the CH/JSXZ/2015 strain of PEDV by the phylogenetic tree construction method. The nucleotide and the amino acid sequences of the CH/JSXZ/2015 strain were compared with the corresponding sequences of PEDV strains deposited in the GenBank database. The PEDV strains used in this study are shown in Table 3. The sequences of 69 complete genomes and their fully sequenced S and ORF3 of PEDV strains were used for sequence alignments and phylogenetic analyses. Furthermore, we aligned nucleotide sequences of full-genomes, ORF3 genes, and S genes of PEDV strains by using the ClustalX 2.0 program [17].

### 2.7. Recombination Analysis

RDP5 software (Recombination Detection Programv 5.05 [18]) was used to identify recombination in the PEDV CH/JSXZ/2015 and other PEDVs.

## 3. Results

### 3.1. Genome-Wide Gene Comparison (Gene Circle Diagram)

**Figure 1:**
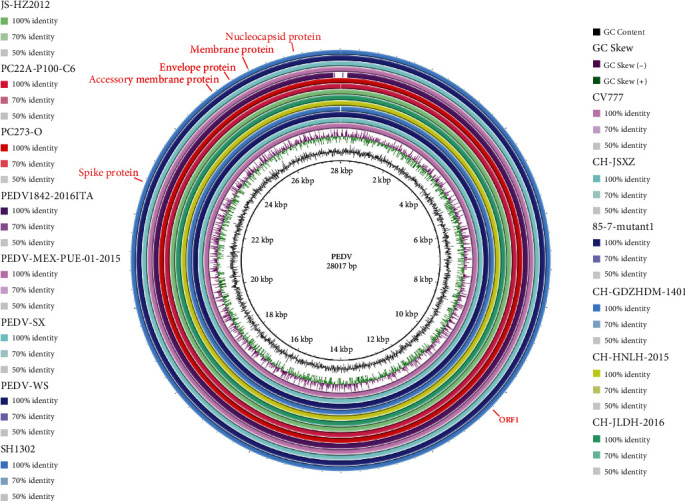
Genome-wide comparison of CH/JSXZ/2015 (CH-JSXZ) with different isolates available in GenBank. We selected representative strains from each region to compare the whole genome with the PEDV CV777 strain as a reference.

We selected 13 representative strains from each region to compare the whole genome with the PEDV CV777 strain as a reference. The genome of CH/JSXZ/2015 (JSXZ) did not show artificial gene deletion of common PEDV vaccine strains, and there was no obvious evidence of other gene sources, and the overall structure was similar to other isolates (Figure 1).

### 3.2. Complete Genomic Characterization of PEDV CH/JSXZ/2015 Strain

The genome of CH/JSXZ/2015 (JSXZ) is 28,044 nucleotides (nt) in length, excluding the poly(A) tail, with a G + C content of 42% and an A + T content of 58%. The genomic organization of the virus was in the following order: 5′ untranslated region (UTR), nucleotide 1–292; replicase polyprotein, nucleotide 293–12,646 for ORF1a and nucleotide 12,601–20,637 for ORF1b; spike (S) protein, nucleotide 20,634–24,794; ORF3, nucleotide 24,794–25,468; envelope (E) protein, nucleotide 25,449–25,679; membrane (M) protein, nucleotide 25,687–26,367; nucleocapsid (N), nucleotide 26,379–27,704; and 3′UTR, nucleotide 27,705–28,044.

Interestingly, compared with CH/JLDH/2016, JSXZ had a single amino acid insertion in the S gene (^1197^T). The genome information of JSXZ will be conducive to understanding the evolutionary characteristics and molecular epidemiology of PEDV in China.

### 3.3. Genome-Wide Sequence Comparison

The complete genome sequences of 70 PEDV strains (Table 3) from different locations and years were compared, and the results revealed that JSXZ had a nucleotide identity of 99.5%–90.1% with 69 other entire PEDV genomes available in GenBank (Table 3). JSXZ showed the highest nucleotide identity (99.5%) with CH/GDZHDM/1401 strain (KR153326.1 and KX016034.1) and the lowest similarity (93.5%) to 85-7-mutant1 strain (KX839247.1). JSXZ shared 93.6% nucleotide similarity with CV777 and 98.9% nucleotide identity with JS-HZ2012 (KC210147.1) strain, which was isolated from the same province with JSXZ, and 98.6% nucleotide similarity to PEDV/MEX/PUE/01/2015 (MH004421.1), PC22A-P100-C6 (KU893871.1), and PC273/O (MG837058.1), respectively.

### 3.4. Phylogenetic Analysis of CH/JSXZ/2015

**Figure 2:**
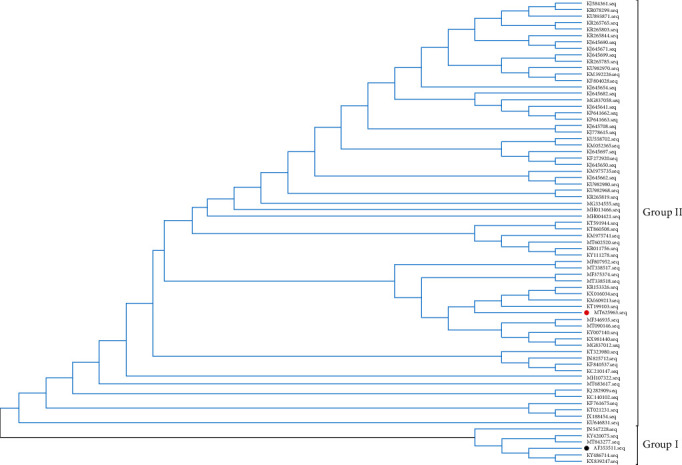
Phylogenetic analysis of the whole genome of PEDV strains (red solid circles indicate PEDV CH/JSXZ/2015, black solid circle indicated PEDV CV777).

To analyze the evolution of PEDV, we performed a phylogenetic tree based on the determined nucleotide sequences of the full-length JSXZ, comparing 69 PEDV strains (Table 3). Interestingly, phylogenetic analysis revealed that these PEDV strains cluster into two groups (Figure 2). Viruses in group Ⅰ includes CV777, PEDV-SX, SH1302, 85-7 mutant1, and 85-7-C40. JSXZ and others belong to Group Ⅱ. JSXZ is closely related to CH/GDZHDM/1401(KR153326.1 and KX016034.1), PEDV-WS, and CH/HNLH/2015.

### 3.5. Characterization and Phylogenetic Analysis of S

S protein is a fibrotic glycoprotein located on the surface of PEDV and the largest structural protein of PEDV [11]. S protein is a key determinant of PEDV invading host cells and a key host antibody response target site [19]. Moreover, it also includes viral neutralizing epitopes that can serve as an important target for vaccine development [20]. Therefore, we conducted a systematic development analysis of S proteins JSXZ with 69 PEDV strains (Table 3). JSXZ S amino acid identity was 90.8%–99.8% compared with other strains.

**Figure 3:**
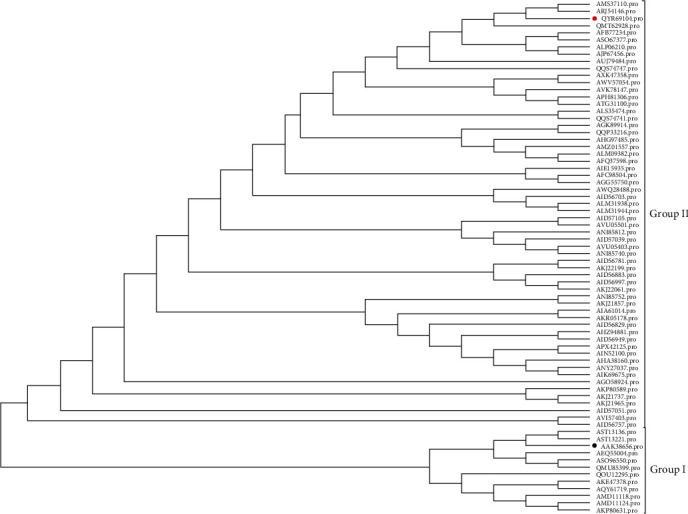
Phylogenetic analysis based on the S protein of PEDV JSXZ strain and 69 historic strains available in GenBank (red solid circles indicate PEDV CH/JSXZ/2015, black solid circle indicated PEDV CV777).

To analyze the evolution of JSXZ, phylogenetic analysis based on 70 PEDV strains S gene (Table 3) was performed. We found a phylogenetic tree cluster into two groups (Figure 3). The result had a similar grouping structure as the tree generated from the PEDV whole genomes. The S of JSXZ belonged to Group Ⅱ. The S of JSXZ is closely related to CH/GDZHDM/1401 (KR153326.1 and KX016034.1).

### 3.6. Characterization and Phylogenetic Analysis of ORF3

ORF3 is the only adjuvant protein of PEDV, located between the S protein and the E protein, composed of 225 amino acids, and the relative molecular mass is about 25 kd*α*. PEDV ORF3 proteins show ion channel activity and enhance the copying ability of viruses [21]. Besides, ORF3 is important in PEDV inhibiting host natural immunity [22]. We compared the amino acid of JSXZ ORF3 identity with 69 PEDV strains (Table 3). The results revealed that JSXZ ORF3 amino acid identity was 91.2%–100.0% compared with other PEDV strains. The results showed that JSXZ ORF3 protein was similar to the remaining strains.

**Figure 4:**
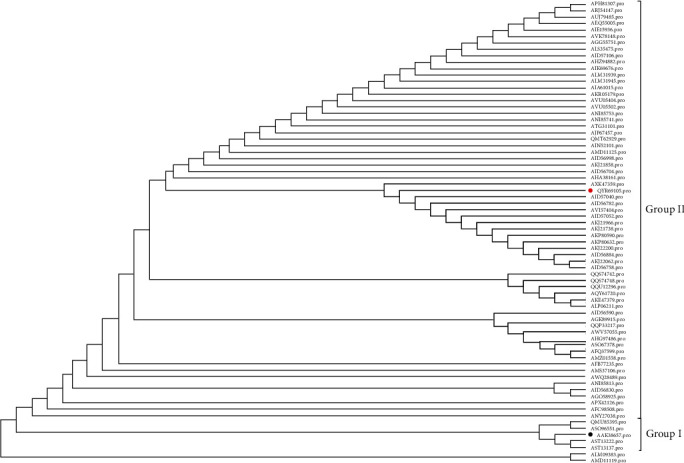
Phylogenetic analysis based on the ORF3 protein of PEDV JSXZ strain and 69 historic strains available in GenBank (red solid circle indicated CH/JSXZ/2015, black solid circle indicated PEDV CV777).

We performed a phylogenetic tree based on the determined nucleotide sequences of ORF3 of JSXZ, comparing 69 PEDV strains ORF3 (Table 3). These PEDV strains cluster into two groups (Figure 4). The result had a similar grouping structure as the tree generated from the PEDV whole genomes and S. The ORF3 of JSXZ belonged to the Group Ⅱ.

### 3.7. Recombination Analysis

**Figure 5:**
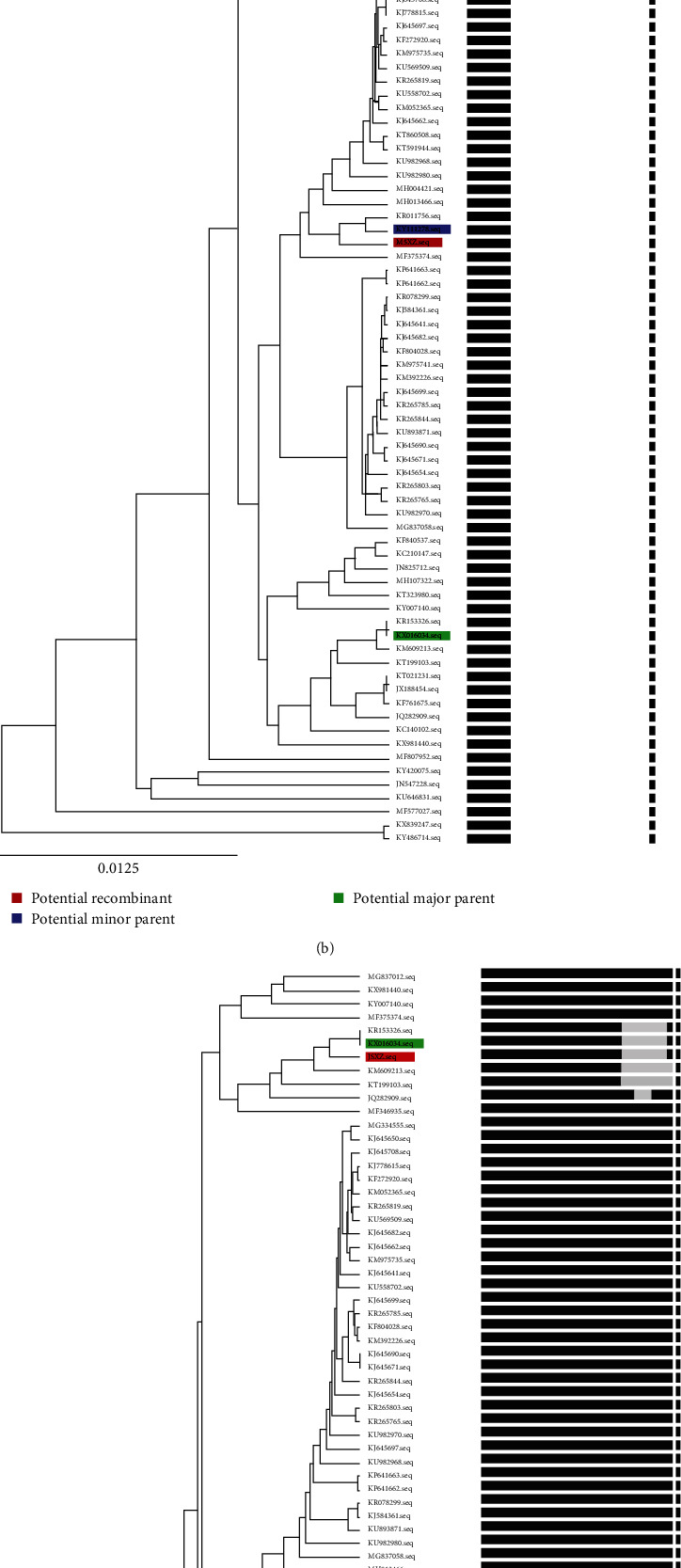
Recombination analysis of PEDV JSXZ strain and 69 historic strains available in GenBank. (a) We used the recombination detection program v5.5, RDP5, to check the recombination. (b) and (c) The evolutionary tree analysis of 1–5530, 28185–28544 and 5531–28184 bp of JSXZ, CH/GDZHDM/1401(KX016034.1) and 1842/2016 ITA (KY111278.1).

Recombination Detection Program v5.5, RDP5 was used to identify recombination in the CH/GDZHDM/1401 (KX016034.1) strain, PEDV 1842/2016 ITA (KY111278.1) and JSXZ, and the position of recombination is 1–5530 and 28185–28544 bp (Figure 5(a)). According to the separation time of the three strains, we speculated that PEDV 1842/2016 ITA isolated by Itay in January 2016 might be obtained by recombination of CH/GDZHDM/1401(KX016034.1) isolated by China in 2014 and JSXZ isolated by China in 2015. According to the results of evolutionary tree analysis (Figures 5(b) and 5(c)), we revealed that 5531–28184 bp of JSXZ are highly homologous to CH/GDZHDM/1401 (KX016034.1), while 1–5530 and 28185–28544 bp of JSXZ are highly homologous to 1842/2016 ITA (KY111278.1).

## 4. Discussion

As the virus continues to evolve and mutate, PEDV is causing more and more damage to the pig herd, as well as causing huge losses to the global pig economy. The early clinical detection diagnosis of PEDV plays a key role in timely and effectively controlling virus dissemination. Vaccination and feedback are the main control measures against PEDV, but these methods do not eradicate and block PEDV effectively [23–25].

In this study, we amplify the whole genome of PEDV from intestinal contents from PED-affected pigs in Jiangsu, China. The complete genome of PEDV CH/JSXZ/2015 (JSXZ) was sequenced and then submitted to GenBank. To provide insight into understanding the genetic, phylogenetic, recombination, and current epidemiological status of PEDV, JSXZ was compared with CV777 and other PEDV strains.

The results showed that the homology between the entire genomic nucleotide sequences of PEDV JSXZ and CV777 was 93.6%. Next, we explored the homology of the S protein and ORF3 proteins of PEDV JSXZ and CV777. The homology of the ORF3 protein between PEDV JSXZ and CV777 was 98.5%. However, the S protein's amino acid sequence identity was 92.4% between PEDV JSXZ and CV777. Besides, the complete genome sequences of PEDV from different locations and years were compared, and the results revealed that JSXZ had the highest nucleotide identity (99.5%) with CH/GDZHDM/1401 strain and the lowest similarity (93.5%) to 85-7-mutant1 strain. The deduced amino acid sequence of JSXZ S protein was compared with 69 historic PEDV strains. The CH/GDZHDM/1401 strain identified the highest nucleotide identity; the lowest was 90.8% compared with 85-7-C40 and 85-7-mutant1 strains. The deduced amino acid sequence of JSXZ ORF3 protein was compared with 69 historic PEDV strains. PEDV JSXZ ORF3 protein is extremely high in most historic strains. The lowest nucleotide identity of ORF3 was 91.2% compared with the YN90 strain. In the phylogenetic tree of the whole genome, the phylogenetic trees based on the whole genome, S and ORF3 were similar. JSXZ is closely related to CH/GDZHDM/1401. In addition, we revealed that the CH/GDZHDM/1401 (KX016034.1) strain, PEDV 1842/2016 ITA (KY111278.1), and JSXZ had recombination, and the position of recombination was 1–5530 and 28185–28544 bp. 5531–28184 bp of JSXZ are highly homologous to CH/GDZHDM/1401 (KX016034), while 1–5530 and 28185–28544 bp of JSXZ are highly homologous to 1842/2016 ITA (KY111278). PEDV JSXZ caused severe diarrhea, vomiting, and dehydration in piglets, leading to a 60% mortality rate at one swine farm from Xuzhou, Jiangsu Province in China. PEDV JSXZ belongs to Group Ⅱ and is a variant strain. Feedback may be the cause of PEDV recombination and increased virulence. Therefore, feedback should be prohibited in swine farms.

JSXZ showed the highest nucleotide identity (99.5%) with the CH/GDZHDM/1401 strain. JSXZ is closely related to the CH/GDZHDM/1401 strain based on the phylogenetic trees. The CH/GDZHDM/1401 (KX016034.1) strain, PEDV 1842/2016 ITA (KY111278.1), and JSXZ had recombination, and the position of recombination was 1–5530 and 28185–28544 bp. This study might provide a reference for the trend of popularity and evolution of PEDV.

## Figures and Tables

**Table 1:** The reagents were used in the paper.

Reagents name	Company	Country
Trizol	Vazyme Biotech	Nanjing, China
TransScript First-Strand cDNA Synthesis SuperMix	Vazyme Biotech	Nanjing, China
Taq enzyme	Vazyme Biotech	Nanjing, China
T-Vector pMD19	Takara Biotech	Dalian, China
AgaroseG-10	Biowest Biotech	Loire Valley, France

**Table 2:** Primers for the amplification of the PEDV genomic fragments.

Primer name	Sequence (5′to 3′)	Product size (bp)
5UTR-F	ACTTAAAAAGATTTTCTATCTACGGATAG	291
5UTR-R	GCCGACAGTTACTGGTTTCA	
ORF1a-1F	ACTAGACAAACAGCCTTCCTCCG	2005
ORF1a-1R	CCTCTCACAACGCAGCTGTAAGTTTTAT	
ORF1a-2F	ATACCTGATTTTCCTATGCCTGTGGC	2127
ORF1a-2R	AACAAGGTCACTAAAGTCTCCTTGGT	
ORF1a-3F	CTGTATTGGAGCCTGTTGTCAAACC	2083
ORF1a-3R	CAGAGACCCAAAATGCTAAGGATGTACA	
ORF1a-4F	ACGCTACAAAGCTGTTAGACACTATG	2025
ORF1b-3R	CGCACTATTGTAACAAGTAGCAACCTTAC	
ORF1b-4F	CTAATTTTTGTGTTGTGGGCTGGTG	2036
ORF1b-4R	TCACTGGCCTGAAGTTGCGGATAAA	
ORF1b-5F	GCATTCTTAAATCGTTGGATTTGAGCG	3032
ORF1b-5R	CCGCCTAAAATTAGTGTTAGCTGAGC	
S1-F	GAATGGTAAGTTGCTAGTGCG	1018
S1-R	ATTAAACCTCAGAGCCTCTGG	
S2-F	CATGGTAAGGTGGTTTCCAACC	1045
S2-R	GGGACGTAAACTTAACACCACTACC	
S3-F	GGACAGTAATTGCCCTTTCACCT	1035
S3-R	CCAAGGCCATTAGTAACCACT	
S4-F	AATGTGCTGGGTGTTTCTGTG	1104
S4-R	ATCTGGGATTACTTCTGGTAGTT	
S5-F	ACCAGGTGACTTTGTAAATGTT	800
S5-R	GCGTTTTGCTGTCATCTT	
ORF3-F	GGTCCTAGACTTCAACCTTA	779
ORF3-R	AGCAGGAAAAAGAGTACGAA	
EMN-F	GTCTTTTCGCAACATCAAATTGTTGGC	2100
EMN-R	ACAAGAAGCTCAACATTTGGATCAGAC	
3UTR-F	ACCAAATGTTGCAGCATTGC	683
3UTR-R	GTGTATCCATATCAACACCGTCA	

**Table 3:** Nucleotide and amino acid sequence identity (%) of different PEDV CH/JSXZ/2015 genome regions compared with the other strains.

	CH/JSXZ/2015							
	Strain	GenBank accession no.	Location	Date	Genome	S aa	ORF3 aa
1	JSXZ	MT625963.1	China	2015	100.0	100.0	100.0
2	CV777	AF353511.1	Switzerland	2001	93.6	92.4	98.5
3	85–7-C40	KY486714.1	China	2017	93.6	90.8	96.1
4	85-7-mutant1	KX839247.1	China	2016	93.5	90.8	95.4
5	AH2012/12	KU646831.1	China	2016	96.9	97.4	96.9
6	AJ1102	JX188454.1	China	2012	98.0	98.2	97.3
7	B5-HB2017	MF807952.1	China	2017	98.5	98.0	100.0
8	BJ−2011–1	JN825712.1	China	2011	98.9	99.0	99.6
9	CH/FJND−3/2011	JQ282909.1	China	2011	98.2	97.2	99.6
10	CH/FJZZ−9/2012	KC140102.1	China	2012	98.2	98.1	99.6
11	CH/GDZHDM/1401	KR153326.1	China	2014	99.5	99.8	100.0
12	CH/GDZHDM/1401	KX016034.1	China	2014	99.5	99.8	100.0
13	CH/HNLH/2015	KT199103.1	China	2015	99.2	99.4	99.6
14	CH/HNZZ47/2016	KX981440.1	China	2016	98.9	98.2	100.0
15	CH/JLDH/2016	MF346935.1	China	2017	98.8	98.8	96.4
16	CH/JXJA/2017	MF375374.1	China	2017	99.0	99.1	100.0
17	CH/S	JN547228.1	China	2011	94.2	91.1	100.0
18	CH/YNKM−8/2013	KF761675.1	China	2013	98.2	98.3	98.2
19	CH/ZJCX−1/2012	KF840537.1	China	2013	98.9	99.3	100.0
20	CHN/SH−2016−4/2016	MG837012.1	China	2018	99.1	98.7	100.0
21	CO/P14/IC	KU558702.1	America	2016	98.8	98.1	98.9
22	FR/001/2014	KR011756.1	France	2015	98.1	95.1	99.6
23	GDS23	MH107322.1	China	2018	98.8	98.6	98.2
24	JS-HZ2012	KC210147.1	China	2012	98.9	99.1	100.0
25	LNCT2	KT323980.1	China	2015	98.9	98.6	100.0
26	MEX/104/2013	KJ645708.1	America	2014	98.8	99.1	100.0
27	NPL-PEDv/2013	KJ778615.1	America	2014	98.8	99.1	100.0
28	OH8593−14	KP641662.1	America	2015	98.8	98.7	100.0
29	OH9097−14	KP641663.1	America	2015	98.8	98.6	100.0
30	OH15962	KJ584361.1	America	2014	98.7	99.1	100.0
31	PC21A	KR078299.1	America	2015	98.7	99.2	100.0
32	PC22A-P100-C6	KU893871.1	America	2016	98.6	98.1	98.7
33	PC273/O	MG837058.1	America	2018	98.6	99.2	99.6
34	PEDV 1842/2016 ITA	KY111278.1	Italy	2016	97.9	94.7	99.6
35	PEDV/MEX/PUE/01/2015	MH004421.1	Mexico	2018	98.6	98.5	100.0
36	PEDV/MEX/QRO/02/2017	MH013466.1	Mexico	2018	98.6	98.7	100.0
37	PEDV/USA/Minnesota125/2015	KU982980.1	America	2016	98.7	98.5	99.2
38	PEDV/USA/NorthDakota93/2015	KU982970.1	America	2016	98.7	99.1	100.0
39	PEDV/USA/Oklahoma133/2015	KU982968.1	America	2016	98.8	98.5	100.0
40	PEDV-LNsy	KY007140.1	China	2016	99.0	98.5	100.0
41	PEDV-SX	KY420075.1	China	2017	94.0	92.3	98.6
42	PEDV-WS	KM609213.1	China	2014	99.3	99.4	100.0
43	TC PC168-P2	KM392226.1	America	2014	98.7	99.1	100.0
44	USA/2014/IL/20697 P7	KT591944.1	America	2015	98.5	95.0	95.1
45	USA/Colorado/2013	KF272920.1	America	2013	98.9	99.2	99.6
46	USA/IL20697/2014 Passage 5	KT860508.1	America	2015	98.5	95.0	100.0
47	USA/Illinois98/2013	KJ645690.1	America	2014	98.7	99.0	100.0
48	USA/Illinois259/2014 from USA	KR265785.1	America	2015	98.8	99.1	100.0
49	USA/Indiana34/2013&America	KJ645641.1	America	2014	98.8	98.9	100.0
50	USA/Iowa/18984/2013	KF804028.1	America	2013	98.7	99.1	100.0
51	USA/Kansas46/2013	KJ645650.1	America	2014	98.9	99.0	100.0
52	USA/Kansas431/2014 from USA	KR265819.1	America	2015	98.9	98.9	100.0
53	USA/Minnesota76/2013	KJ645671.1	America	2014	98.7	99.0	100.0
54	USA/Minnesota90/2013	KJ645682.1	America	2014	98.8	99.1	99.6
55	USA/Missouri373/2014 from USA	KR265844.1	America	2015	98.7	98.7	100.0
56	USA/MO/2014/03293	KM975741.1	America	2014	98.2	94.9	100.0
57	USA/NC/2013/35140	KM975735.1	America	2014	98.9	99.0	100.0
58	USA/Nebraska287/2014 from USA	KR265765.1	America	2015	98.7	99.0	100.0
59	USA/Nebraska288/2014 from USA	KR265803.1	America	2015	98.7	99.0	100.0
60	USA/NorthCarolina66/2013	KJ645662.1	America	2014	98.9	99.1	99.6
61	USA/Ohio123/2014	KJ645699.1	America	2014	98.8	99.1	100.0
62	USA/OK10240−8/2017	MG334555.1	America	2017	98.8	99.0	100.0
63	USA/Tennesse56/2013	KJ645654.1	America	2014	98.7	98.7	100.0
64	YN90	KT021231.1	China	2015	98.1	97.8	91.2
65	PEDV/Pig-wt/ESP/Calaf-1/2014	MT602520.1	Spain	2020	98.0	95.3	99.1
66	HNAY2016	MT338518.1	China	2021	99.0	99.5	99.2
67	CH/HNXX/2016	MT338517.1	China	2021	98.5	97.9	98.2
68	CH/SXWS/2018	MT090146.1	China	2020	99.1	99.2	100.0
69	SH1302	MT843277.1	China	2020	94.0	98.6	92.4
70	MSCH	MT683617.1	China	2021	98.7	97.9	98.2

Abbreviations: aa, amino acid.

## Data Availability

The data that support the findings of this study are openly available in the National Center for Biotechnology Information at https://www.ncbi.nlm.nih.gov/nuccore/MT625963.1/, reference number MT625963.1.
